# Wafer-Level Transfer of GaN-on-Si Light-Emitting Devices via SiO_2_–SiO_2_ Direct Bonding: Strain Evolution and Optoelectronic Performance

**DOI:** 10.3390/mi17050607

**Published:** 2026-05-15

**Authors:** Siyi Zhang, Shuhan Zhang, Qian Fan, Xianfeng Ni, Xing Gu

**Affiliations:** Institute of Next Generation Semiconductor Materials, Southeast University, Suzhou 215123, China; 220234936@seu.edu.cn (S.Z.); shuhanzhang@seu.edu.cn (S.Z.); 103200035@seu.edu.cn (Q.F.); 103200036@seu.edu.cn (X.N.)

**Keywords:** GaN-on-Si, Micro-LEDs, SiO_2_–SiO_2_ direct bonding, substrate transfer

## Abstract

GaN-on-Si light-emitting devices have been widely studied in the field of opto-electronics, while their optical performance and characterization accessibility are severely limited by the strong visible light absorption of the native silicon substrate. Conventional substrate transfer technologies often suffer from inherent thermal, optical, or mechanical bottlenecks. In this study, we developed a robust wafer-level substrate transfer strategy for 8-inch green GaN-on-Si light-emitting device wafers, utilizing a hybrid planarization process combined with SiO_2_–SiO_2_ direct bonding. The hybrid planarization precisely eliminated the 900 nm macroscopic steps, achieving sub-nanometer surface roughness for high-yield wafer bonding. We systematically investigated the physical evolution during substrate removal. Results indicate that the removal of the thick native silicon and high-stress buffer layers effectively released the additional in-plane biaxial compressive stress within the multiple quantum wells (MQWs), thereby mitigating the quantum-confined Stark effect (QCSE). Benefiting from the elimination of the light-absorbing silicon substrate and the incorporation of a built-in back-surface reflector (BSR), the transferred devices achieved a remarkable 1.9-fold enhancement in relative optical performance, albeit with an inherent trade-off of increased reverse leakage current while preserving basic diode functionality. Furthermore, optothermal dynamic analysis at high injection levels suggests a potential localized thermal bottleneck at the thick SiO_2_ bonding interface, where a hypothesized heat-induced spectral red shift may counteract the carrier-screening blue shift. This work provides a feasible wafer-level substrate transfer process for GaN-on-Si devices and offers systematic experimental insights into stress relaxation and optothermal behaviors during the substrate transfer process.

## 1. Introduction

Micro-light-emitting diodes (Micro-LEDs) have been widely investigated for advanced optoelectronic applications, owing to their outstanding advantages in brightness, contrast, response speed, and long-term stability under harsh environments [1,2,3,4]. For the fabrication of high-brightness blue and green light-emitting devices, the InGaN/GaN material system is the most mature and mainstream choice. Regarding the epitaxial growth substrate, compared to traditional sapphire substrates with inherent limitations in thermal conductivity and electrical insulation, silicon (Si) substrates have been widely recognized as a promising platform for GaN-based optoelectronic devices. This is primarily attributed to their mature large-scale wafer manufacturing processes, low material cost, excellent thermal conductivity, and inherent compatibility with complementary metal-oxide-semiconductor (CMOS) manufacturing lines [5,6].

Despite the above advantages of GaN-on-Si technology, the narrow bandgap of silicon renders it a strong absorption medium for visible light. Consequently, a large portion of photons emitted downward from the active region is absorbed by the native silicon substrate, leading to a drastic reduction in light extraction efficiency (LEE) of the devices [7]. Furthermore, the opacity of the silicon substrate completely blocks the backside optical access, making it difficult to perform accurate optoelectronic characterization and failure analysis of the device arrays from the backside. Therefore, to address the above issues, substrate lift-off and transfer technologies are required, which refer to detaching the as-grown GaN epitaxial layers from the native silicon substrate and transferring them onto a target carrier substrate [8].

Among the various substrate transfer technologies explored to date, micro-transfer printing or adhesive bonding based on organic polymers has been widely adopted due to its broad process window and high tolerance for surface topography [9,10,11]. However, when used as the bonding medium, these organic polymer layers have inherently poor thermal conductivity, which will introduce a large thermal resistance at the bonding interface and lead to accelerated device degradation under high-current or high-frequency driving conditions. More critically, the significant refractive index mismatch between organic materials, high-index GaN, and the carrier substrate will trigger total internal reflection (TIR) at the interface, which further reduces the light extraction efficiency [12,13,14].

Recently, flip-chip bonding (FCB) has been widely studied for the assembly of Micro-LED arrays, which achieves electrical and mechanical interconnection between the device and the driver substrate via metallic micro-bumps (e.g., Indium and Tin) [15,16,17]. While FCB shows good feasibility for small-scale array assembly, it faces several inherent bottlenecks when applied to large-area wafer-level transfer. First, the transfer yield of flip-chip processes is difficult to meet the requirements of high-pixel-density device arrays. Second, the large mismatch in the coefficient of thermal expansion (CTE) between silicon and GaN will easily cause micro-bump cracking and interfacial failure during the high-temperature bonding process [18,19]. Most crucially, in conventional flip-chip architectures, the silicon substrate is often retained, which means the aforementioned issues of light absorption and characterization obstruction remain unresolved.

In contrast, SiO_2_–SiO_2_ wafer-level direct bonding presents distinct advantages. By eliminating the need for any intermediate adhesive layers, this technology not only ensures superior mechanical reliability but also mitigates optical discontinuities and air gaps at the interface. This establishes a continuous and optimized optical medium channel for the efficient extraction of photons at the micro and nanoscales [20,21,22]. However, SiO_2_–SiO_2_ direct bonding imposes stringent requirements on surface morphology, where achieving void-free bonding across an entire wafer is predicated on maintaining a sub-nanometer surface roughness [23,24].

Direct wafer bonding has been explored in GaN-based materials and device platforms. Ryu et al. demonstrated the transfer of AlGaN/GaN epilayers grown on Si(111) onto Si(100) wafers by direct wafer bonding, followed by selective dry etching to fabricate thin-body N-face GaN-on-insulator wafers for HEMT applications [25]. Dragoi et al. reported GaN-engineered substrates based on SiO_2_-assisted plasma-activated bonding and oxide-free covalent bonding, highlighting the flexibility of wafer bonding for integrating GaN with different target substrates [26]. In addition, the electrical characteristics of SiO_2_/GaN interfaces have been shown to be strongly affected by Ga–OH-related interfacial states, emphasizing the importance of interface chemistry in GaN/oxide systems [27]. However, these studies mainly focused on transistor-oriented N-face GaN transfer, engineered GaN substrates, or electrically active SiO_2_/GaN interfaces. Wafer-level transfer of fully processed GaN-on-Si light-emitting devices, especially after the formation of mesa structures and thick metal electrodes, remains less explored.

Different from the transfer of bare epitaxial layers or transistor-oriented GaN structures, the wafers used in this work had already undergone front-side LED fabrication. The mesa etching and Ni/Al/Ti p-metal deposition introduced macroscopic steps of approximately 900 nm. To overcome the severe geometric undulations introduced by the front-end fabrication process, this study developed a hybrid planarization strategy and successfully achieved wafer-level bonding and substrate transfer of 8-inch GaN-on-Si green light-emitting device wafers. To systematically explore the fundamental physical evolution during the transfer process, devices with a mesa diameter of 200 μm were adopted as representative test vehicles in this study. Although their size exceeds the definition of conventional Micro-LEDs, they were fabricated with the full front-end process flow of Micro-LEDs, and provide a robust and representative platform to clearly analyze the complex stress, electrical, and optothermal dynamic behaviors during the bonding and transfer process without the interference of size effects.

This work conducted multidimensional analyses on the evolution of the mechanical, optical, and electrical properties of the devices before and after transfer. The study first revealed the stress evolution law during the substrate thinning process, confirming that the removal of the massive bulk silicon and high-stress buffer layer caused severe fluctuations in macroscopic wafer warpage while realizing the steady relaxation of the additional in-plane compressive stress in the active region, which effectively mitigates the quantum-confined Stark effect (QCSE). The impact of substrate removal and thermal annealing on the electrical characteristics of the devices was also evaluated, confirming that the core p-n junction rectification characteristics remained robust despite an inevitable increase in process-induced leakage current. More importantly, the luminescence test results verified the significant optical benefits brought by substrate transfer: by eliminating the strongly light-absorbing native silicon substrate and introducing a built-in metal Back-Surface Reflector (BSR), a 1.9-fold increase in luminous intensity was achieved at low injection currents. These detailed optoelectromechanical analyses provide solid process verification and experimental reference for the substrate transfer of GaN-on-Si optoelectronic devices.

## 2. Materials and Methods

**Figure 1:**
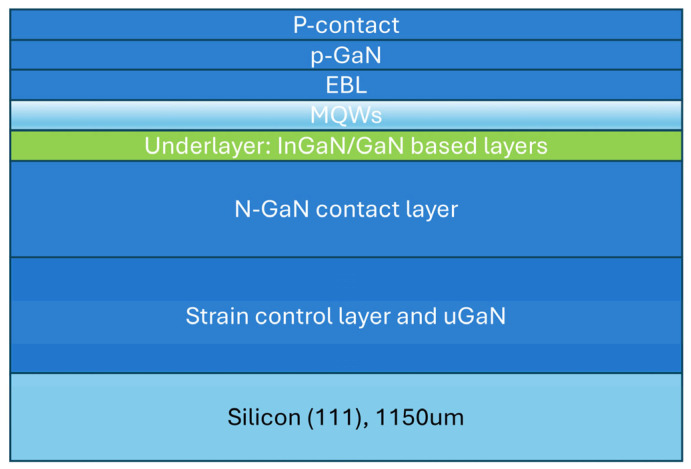
Schematic diagram of the epitaxial structure for green Micro-LEDs.

The 8-inch green GaN-on-Si (111) epitaxial wafers used in this study were grown via metal–organic chemical vapor deposition (MOCVD) by Suzhou Hanhua Semiconductors Ltd. (Suzhou, China). As illustrated in Figure 1, the epitaxial structure primarily consists of the following: an AlN/AlGaN strain-control buffer layer to mitigate the mismatch in lattice constants and thermal expansion coefficients, a high-crystalline-quality unintentionally doped GaN (u-GaN) layer, a Si-doped n-GaN contact layer (approximately 1 μm thick), an InGaN/GaN-based strain-release underlayer, an InGaN/GaN multiple quantum well (MQW) active light-emitting region with a peak emission wavelength centered around 520 nm, and a Mg-doped p-GaN hole injection layer.

**Figure 2:**
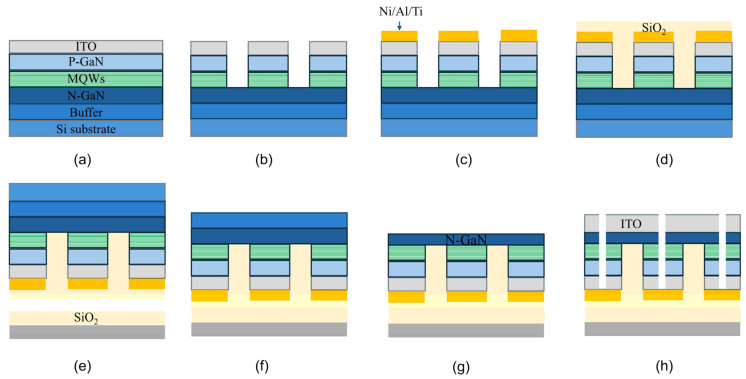
Schematic of the fabrication process: (**a**) deposition and thermal annealing of the 100 nm-thick ITO transparent conductive layer; (**b**) mesa etching to define the light-emitting area; (**c**) deposition of the p-metal (Ni/Al/Ti) on the mesa; (**d**) deposition of a 1.5 μm SiO_2_ layer covering the entire structure, followed by planarization via CMP; (**e**) wafer-level direct bonding to a silicon carrier wafer with a 300 nm SiO_2_ layer; (**f**) complete removal of the native silicon substrate; (**g**) thinning of the N-face nitride materials to remove the buffer layers and expose the n-GaN; (**h**) deposition of N-face ITO and fabrication of through-vias.

**Figure A1:**
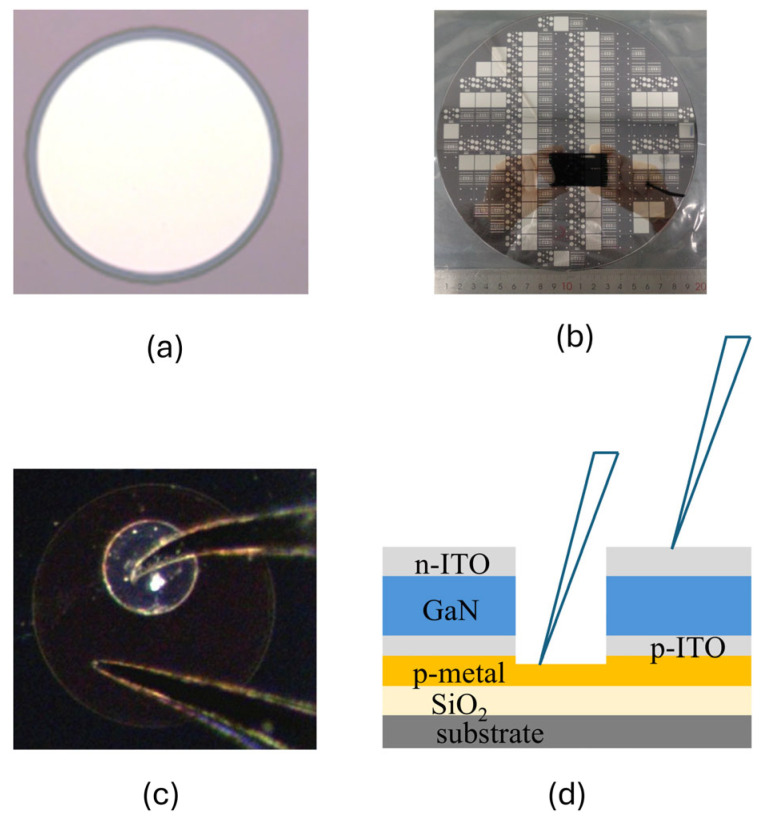
Real morphology of front-side LED structures and EL measurement configuration: (**a**) Optical microscopy image of the fabricated circular LED mesa. (**b**) Representative optical photograph of an 8-inch wafer with fabricated LED device structures. (**c**) Optical photograph of the actual EL testing process. (**d**) Cross-sectional schematic diagram illustrating the EL measurement setup.

Figure 2 illustrates the overall fabrication process flow. To verify the wafer-level, ultra-high-yield bonding and non-destructive substrate lift-off, front-side fabrication processes for Micro-LEDs were performed on the epitaxial wafers. As shown in Figure 2a, a 100 nm thick indium tin oxide (ITO) layer was first deposited onto the p-GaN surface, followed by rapid thermal annealing (RTA) at 600 °C for 5 min to form a high-quality ohmic contact. Subsequently, as depicted in Figure 2b, circular mesa structures with a diameter of 200 μm were defined via photolithography and a Cl_2_-based inductively coupled plasma (ICP) etching process. Following this, a Ni/Al/Ti composite multilayer metal stack with thicknesses of 100 nm/400 nm/20 nm was sequentially deposited onto the ITO surface of the mesas via electron beam evaporation, as shown in Figure 2c. This metal layer serves not only as the p-contact interconnection electrode but also acts as a BSR in the final transferred device architecture. The real morphology corresponding to the processing stage shown in Figure 2c is provided in Figure A1a,b. Figure A1a shows the optical microscope image of the fabricated circular LED mesa structure after Ni/Al/Ti p-metal deposition, and Figure A1b presents a representative optical photograph of the 8-inch wafer.

The aforementioned etching and the introduction of the thick multilayer metal electrodes resulted in macroscopic steps of up to 900 nm. To eliminate these steps and prepare a high-quality interface for bonding, a hybrid planarization strategy combining selective etching and multiple rounds of chemical–mechanical polishing (CMP) was employed in this study. As shown in Figure 2d, an approximately 1.5 μm thick SiO_2_ layer was deposited over the entire device wafer surface using plasma-enhanced chemical vapor deposition (PECVD). Due to the high conformality of the PECVD process, the underlying 900 nm macroscopic steps were faithfully replicated on the oxide surface, resulting in significant topographic undulations. Subsequently, precise overlay lithography and dry etching were utilized to selectively remove these protruding oxide features. Finally, the wafer surface underwent multiple CMP treatments to achieve a sub-nanometer smooth surface (Ra < 0.21 nm) suitable for direct bonding.

Following planarization, the wafers were ready for bonding. An 8-inch silicon wafer with a 300 nm oxide layer grown on its surface was selected as the receptor carrier wafer. Both the planarized Micro-LED wafer and the silicon carrier wafer were subjected to an optimized N_2_/O_2_ mixed plasma activation treatment at room temperature to achieve a highly hydroxylated surface. Subsequently, a high-precision alignment system was used to accurately align and pre-bond the two wafers in a vacuum environment, as illustrated in Figure 2e. The pre-bonded wafer pair was then placed in a vacuum annealing furnace and annealed at a constant temperature of 300 °C for 3 h to form a dense SiO_2_–SiO_2_ covalent bond network.

After bonding, a combined thinning process was performed on the native thick silicon substrate. First, back-grinding was used to rapidly and completely remove most of the bulk native silicon, as shown in Figure 2f. Subsequently, highly selective deep ICP etching was utilized to completely clear the residual silicon and the underlying high-stress buffer layers, achieving precise thinning of the n-GaN face and completing the non-destructive, wafer-level transfer of the epitaxial layers, as illustrated in Figure 2g. After removing the native substrate, exposing the N-face, and completing the thinning process, a 200 nm thick ITO transparent conductive layer was grown on the n-GaN surface, as shown in Figure 2h. To facilitate optoelectronic characterization, probe-access vias with a diameter of 80 μm were defined using photolithography and ICP etching, providing localized access to the buried p-metal electrodes. The optical morphology of the fabricated mesa and the detailed cross-sectional schematic of the probing configuration are provided in Figure A1c,d of Appendix A. During the electroluminescence (EL) measurements, a micro-probe station (with a typical probe tip diameter of 10–20 μm) was employed to establish the electrical interconnection: one probe was precisely positioned through the via to contact the underlying p-electrode, while the complementary probe was biased against the n-face ITO transparent conductive layer, thereby completing a robust vertical current injection path. The current density and integrated optical intensity were normalized to the active LED mesa area, which excluded the vias.

## 3. Results and Discussion

### 3.1. Hybrid Planarization and Wafer-Level Transfer

Achieving high-precision, high-yield wafer-level direct bonding is critically dependent on achieving sub-nanometer surface roughness and the complete elimination of macroscopic topographic fluctuations. As previously described, the mesa etching and the deposition of thick multilayer p-metal electrodes inevitably introduced a severe macroscopic step height of approximately 900 nm on the wafer surface. To overcome this significant geometric obstacle, a hybrid planarization strategy was systematically developed, and its evolution was closely monitored in this study.

**Figure 3:**
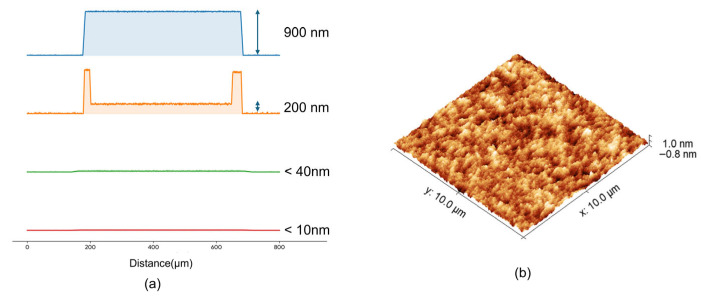
Surface morphology characterization of the planarization process: (**a**) the evolution of surface morphology at different stages of the hybrid planarization; (**b**) 3D AFM image of the final SiO_2_ bonding surface after the second CMP treatment, indicating sub-nanometer roughness.

Figure 3a displays the surface profile evolution recorded by a step profiler at key stages of the planarization process. In the initial state, a distinct step height of 900 nm is observed above the Micro-LED mesa. The subsequently deposited 1.5 μm-thick SiO_2_ layer via PECVD exhibited highly conformal growth characteristics, almost entirely preserving the macroscopic height difference (with a variation of less than 20 nm). Consequently, direct chemical–mechanical polishing (CMP) at this stage would inevitably trigger severe pattern density effects and localized dishing. To circumvent this issue, this study employed a strategy involving overlay lithography combined with selective dry etching to precisely reduce the protruding oxide features. As shown by the second profile in Figure 3a, this dry etching step significantly reduced the steep macroscopic step height.

The pre-planarized surface then underwent the first round of CMP, followed by the conformal deposition of an additional 300 nm SiO_2_ layer to repair any potential residual micro-scratches and provide a fresh, pristine oxide layer for final polishing. The third and fourth profiles in Figure 3a vividly illustrate the progressive elimination of the step height. After the second CMP treatment, a flat macroscopic surface was finally obtained, with the global height variation reduced to a negligible range of 10–20 nm.

Beyond macroscopic flatness, the microscopic surface roughness directly determines the density of the interfacial covalent bonds. Figure 3b displays the three-dimensional (3D) atomic force microscopy (AFM) morphology of the SiO_2_ surface after the second CMP process, acquired over a scanning area of 10 μm × 10 μm with a resolution of 256 × 256 pixels. The 3D topography clearly reveals a uniformly dense surface feature without any visible macro-protrusions, residual particles, or scratches, demonstrating the effectiveness of the hybrid planarization process. The measured root-mean-square roughness (Sq) is 0.200 nm, while the average roughness (Sa) is 0.158 nm, and the maximum peak-to-valley height (Sz) is strictly limited to within 2.791 nm. These sub-nanometer roughness metrics perfectly meet the stringent requirements for spontaneous covalent SiO_2_–SiO_2_ bonding under room-temperature activation, fundamentally ensuring the high yield of the subsequent wafer-level bonding and transfer.

**Figure 4:**
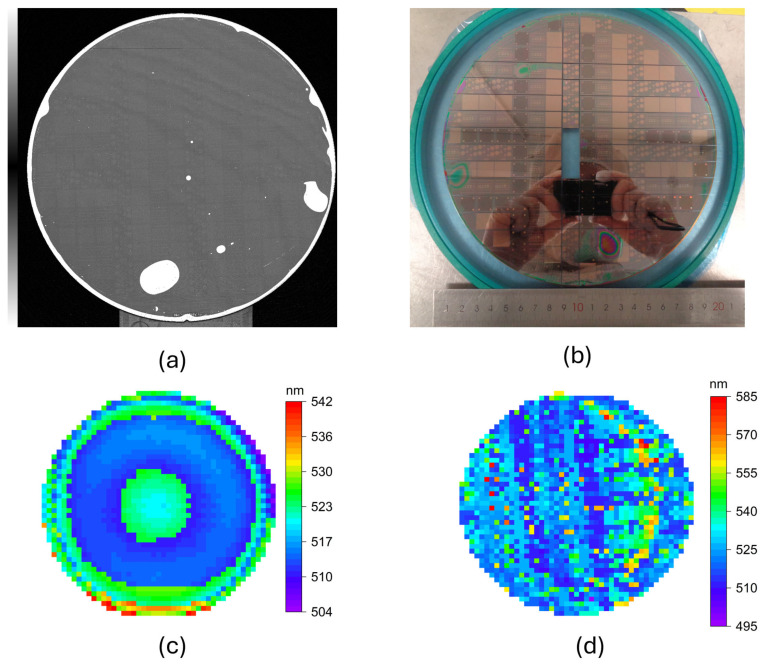
Evidence of the transfer yield at the macroscopic scale: (**a**) Post-bond full-wafer SAM image. (**b**) Normal optical photograph of the wafer after bonding, removal of the native silicon substrate, and n-GaN thinning. Part of the wafer was diced for subsequent experiments, and the remaining portion is shown. (**c**) The PL map of the initial GaN-on-Si epitaxial wafer before deice fabrication. (**d**) The PL map of the post-transfer wafer after SiO_2_–SiO_2_ direct bonding and complete removal of the native bulk silicon substrate.

After the formation of the covalent SiO_2_–SiO_2_ interface, the bonded wafer pair underwent extremely rigorous mechanical grinding and reactive ion etching to completely remove the approximately 1 mm thick native bulk silicon substrate. This etching process was precisely stopped at the AlN/AlGaN buffer layer. Such a harsh thinning process typically introduces enormous shear stress; if the bonding interface were weak, it would immediately trigger massive film delamination or catastrophic fracture. A full-wafer scanning acoustic microscopy (SAM) image of the bonded wafer pair is shown in Figure 4a, and a normal photograph of the wafer after bonding, removal of the native silicon substrate, and n-GaN thinning is provided in Figure 4b. Part of the wafer had been diced for subsequent experiments, so the remaining portion of the actual post-transfer wafer is shown. While the optical photograph provides an intuitive view of the wafer appearance after bonding and substrate removal, the SAM image provides clearer and more quantitative visualization of the bonding quality. Analysis of the SAM image using ImageJ (version 1.54g) identified 11 visible voids and showed that the void-affected area fraction is approximately 4.301%. In addition, the fraction of LED-patterned regions not overlapped by any voids was found to exceed 95%, indicating that the majority of the LED regions on the wafer were structurally preserved after bonding. Additionally, optical and electrical characterization was performed on devices selected from regions without visible bonding defects.

Figure 4c and Figure 4d present the full-wafer PL peak wavelength mappings before and after substrate transfer, respectively. Figure 4c displays the typical concentric circular emission pattern of the initial GaN-on-Si epitaxial wafer, while Figure 4d shows that after complete removal of the native silicon substrate, the 8-inch ultrathin epitaxial film maintained a high degree of overall integrity on the carrier wafer. Combining the SAM images and pl map, it can be concluded that although a small number of macroscopic bubbles or voids can be observed at the edges or in localized areas (which may originate from trace particle contamination or gas trapping during the room-temperature bonding process), they did not propagate to cause catastrophic large-area film peeling, nor did they significantly affect the core active light-emitting region. That macroscopic optical integrity conclusively proves that the sub-nanometer smooth surface achieved through hybrid planarization was successfully transformed into an extremely robust covalent bonding network, capable of withstanding the extreme mechanical stress challenges of the subsequent processes. Furthermore, a careful comparison of the two peak wavelength mappings reveals a global blue shift in the overall emission wavelength after the removal of the silicon substrate. This physical phenomenon will be analyzed in detail in the next section.

### 3.2. Strain Relaxation and Optical Evolution Analysis

**Figure 5:**
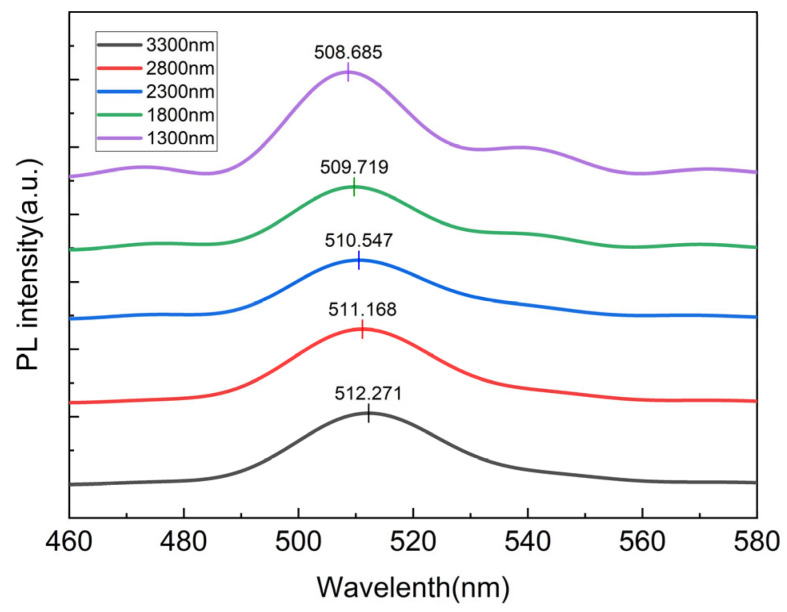
Stacked photoluminescence (PL) spectra of the InGaN/GaN MQWs at different remaining epitaxial thicknesses during the progressive back-thinning process.

The mechanical removal of the massive native silicon substrate and the severely lattice-mismatched buffer layer inevitably triggers a drastic redistribution of internal stress within the GaN epitaxial layer. High-resolution X-ray diffraction (HRXRD) would provide a direct measurement of lattice strain. However, the strain evolution in this study had to be monitored while maintaining the bonded sample as an intact 8-inch wafer during the sequential substrate-removal process. The available XRD system could not directly accommodate the full-size 8-inch bonded wafer. Although cleaving the wafer into small pieces would enable conventional XRD measurements, such a destructive process would partially release the wafer-scale mechanical constraint, introduce edge-related stress relaxation, and prevent continuous characterization on the same wafer during the subsequent thinning steps. Therefore, instead of XRD, we employed a non-destructive characterization strategy combining PL spectroscopy of the MQWs with wafer-scale warpage measurements using a non-contact thickness gauge. In this context, the PL peak shift and FWHM evolution are used as optical indicators of relative strain relaxation in the active region, while the warpage data reflect the macroscopic mechanical response of the bonded wafer stack. It should be specifically noted that due to localized stress modulation and slight process deviations introduced during the wafer bonding process, the global PL emission often exhibits a certain degree of spatial non-uniformity. Therefore, to strictly eliminate the interference of spatial variations and accurately track the intrinsic strain evolution, a fixed representative micro-area near the center of the wafer was selected for continuous in situ PL monitoring after each sequential thinning step in this study. Representative stacked PL spectra recorded during the progressive back-thinning process are shown in Figure 5.

**Figure 6:**
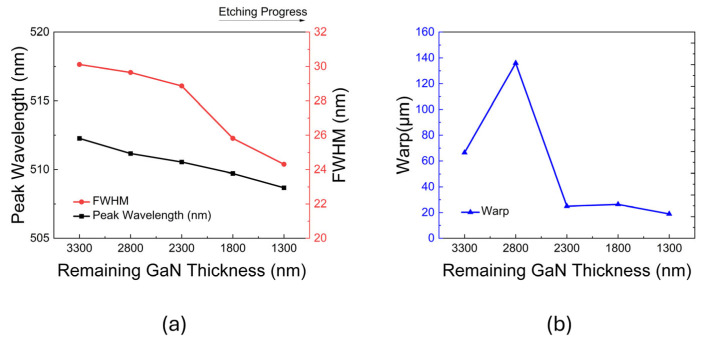
Decoupling of macroscopic structural deformation and microscopic localized strain: (**a**) Evolution of the PL peak wavelength and full width at half maximum (FWHM) as a function of the remaining GaN thickness. (**b**) Corresponding macroscopic wafer warpage measured by a non-contact thickness gauge.

The extracted PL peak wavelength, FWHM, and wafer warpage are summarized in Figure 6a,b. Interestingly, although the microscopic active region exhibited an extremely steady and monotonic strain relaxation, the macroscopic warpage fluctuated violently due to structural asymmetry. Specifically, as shown in Figure 6b, the warpage exhibited a dramatic peak at the remaining thickness of 2.8 μm. Based on the epitaxial design, it is hypothesized that the removal of the highly strained AlN/AlGaN buffer layer at this stage temporarily disrupts the stress balance, potentially causing the observed peak before further strain relaxation. Meanwhile, as shown in Figure 6a, the PL peak wavelength continuously blue-shifted from approximately 514 nm to 511 nm, accompanied by a noticeable narrowing of the full width at half maximum (FWHM). This microscopic evolution is consistent with the progressive release of the additional in-plane biaxial compressive stress within the InGaN/GaN MQWs during the removal of the underlying constraint layers. This strain relaxation is expected to weaken the piezoelectric polarization field, thereby mitigating the quantum-confined Stark effect (QCSE). The previously tilted energy bands consequently recovered to a flatter state, resulting in an increased effective bandgap (wavelength blue shift). Meanwhile, the FWHM narrowing is primarily driven by the homogenization of local strain gradients and the reduced sensitivity of quantized energy levels to interfacial thickness fluctuations within the flattened band structure, which collectively suppresses the inhomogeneous broadening of the emission spectrum.

### 3.3. Electrical Performance Analysis

**Figure 7:**
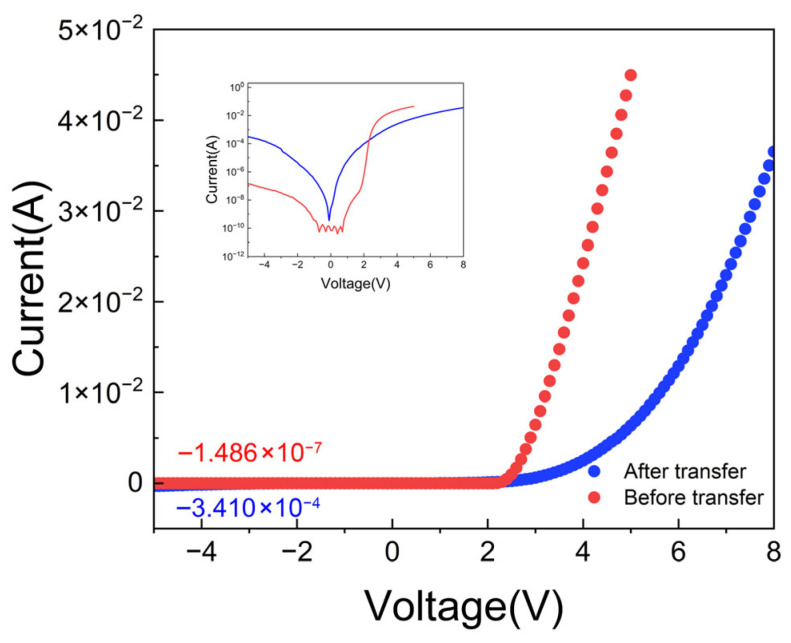
Current–voltage (I–V) characteristics of the Micro-LEDs before (red) and after (blue) the wafer-level transfer.

To evaluate the electrical integrity of the Micro-LEDs after undergoing extreme physical and thermal processes, such as wafer bonding and substrate lift-off, the current-voltage characteristics before and after transfer were compared. As shown in Figure 7 (plotted on both linear and semi-logarithmic axes), although the electrical framework exhibited a certain degree of process-induced degradation, the core rectification characteristics of the p-n junction were successfully preserved. Specifically, in the reverse bias region, the transferred device exhibited a leakage current of approximately 3.1 × 10^−4^ A at 5 V, which is an increase of about three orders of magnitude compared to the pre-transfer baseline of 1.48 × 10^−7^ A. This increased leakage can be primarily attributed to two factors: first, the ICP etching performed to fabricate probe access via inevitably introduced plasma damage and dense surface states on the sidewalls, which act as non-radiative Shockley–Read–Hall (SRH) leakage channels; second, the profound strain relaxation (as evidenced in Section 3.2), combined with thermal annealing at 300 °C, may have activated latent threading dislocations as conductive leakage paths.

Furthermore, in the forward bias region, the turn-on voltage increased from approximately 2.5 V to 3.3 V, accompanied by a decrease in the slope of the I–V curve. This indicates a significant increase in the series resistance of the device, likely related to the slight degradation of the fragile p-GaN/ITO ohmic contact interface due to the thermal budget during the bonding process. To further evaluate device variation, turn-on voltage and reverse leakage current at −5 V were extracted from five devices before transfer and five devices after transfer, as summarized in Table A1. The statistical results show the same general trend as the representative I–V curves in Figure 7: the transferred devices remain functional but exhibit higher turn-on voltage and increased reverse leakage compared with the pre-transfer devices. Overall, the current transfer process successfully preserves the normal diode operation of the devices, allowing further optical-intensity measurements to be performed and thereby enabling evaluation of the effect of substrate transfer. However, for practical Micro-LED applications, this level of increased leakage current (by more than three orders of magnitude) and elevated turn-on voltage requires further optimization. The following electroluminescence analysis is therefore conducted to assess whether the transferred device architecture can still provide optical-performance enhancement despite the observed electrical degradation. Meanwhile, for future practical Micro-LED devices, the balance between optical enhancement and electrical degradation should also be carefully considered.

### 3.4. Optical Enhancement and Thermal Limitations

**Figure 8:**
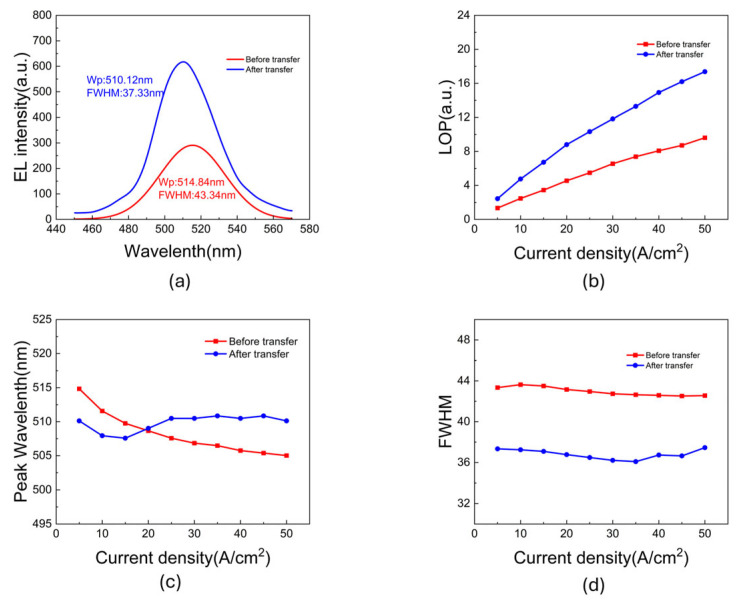
Optoelectronic luminescence characteristics before (red) and after (blue) the transfer: (**a**) EL spectra at a low injection current density of 5 A/cm^2^. (**b**) Integrated EL intensity as a function of injection current density (L-J curves), (**c**) peak wavelength, and (**d**) FWHM evolution versus current density.

To verify the core objective of this wafer-level substrate transfer—namely, improving optical performance by replacing the strongly absorbing silicon substrate—a comprehensive analysis of the electroluminescence (EL) performance was conducted. Figure 8a compares the EL spectra at a low injection current density of 5 A/cm^2^. To ensure a rigorous and fair optical comparison, the baseline spectrum before transfer was mathematically smoothed and fitted to remove the strong Fabry–Pérot (FP) interference fringes originally formed by the GaN/air and GaN/Si interfaces, thereby accurately extracting its intrinsic spontaneous emission envelope.

As shown in Figure 8a, the integrated EL intensity of the transferred device exhibited a remarkable 1.9-fold increase (nearly 100% enhancement) compared to the silicon-based reference device. The approximately 1.9-fold increase in EL intensity indicates improved light extraction in the transferred device architecture. Before transfer, photons emitted toward the substrate side could be absorbed by the native Si substrate. After substrate transfer and Si removal, the Ni/Al/Ti p-metal becomes the backside optical boundary beneath the light-emitting region, where it can partially redirect downward-propagating photons toward the emitting side. Therefore, the observed EL enhancement is attributed to the combined optical effect of removing the absorbing Si substrate and enabling a reflective backside boundary through the transfer process. These two changes occur simultaneously in the present device structure. The reflection efficiency may depend on the specific backside metal stack, and further optimization using different reflector materials will be considered in future work.

Remarkably, as revealed by the macroscopic integrated intensity versus current density curves in Figure 8b, this significant optical dividend is strongly maintained even at higher injection levels. Throughout the tested current range, the emission intensity maintained a growth trend without exhibiting significant efficiency droop. This continuous enhancement proves that within the current operating range, the optical gain provided by substrate transfer dominates the device performance. However, it is essential to objectively evaluate this optical success against the electrical degradation discussed in Section 3.3. While the 1.9-fold optical output enhancement successfully validates the transfer strategy for the current test devices, the accompanying increase in reverse leakage current is a significant process-induced defect. When facing future applications of true Micro-LEDs, such a severe leakage current cannot be ignored, as it would severely compromise overall efficiency and reliability. This inherent trade-off explicitly indicates that although the optical architecture is successfully verified, the current process conditions must be rigorously optimized in future work. Despite the strong growth in macroscopic EL intensity, the evolution of the microscopic spectra—particularly the FWHM and peak wavelength—serves as an extremely sensitive probe revealing the underlying optothermal dynamics. As shown in Figure 8d, the FWHM of the transferred device remained narrower than the pre-transfer baseline across all injection currents. This phenomenon corroborates the PL results in Section 3.2, confirming that substrate lift-off and N-face thinning released the additional stress, thereby weakening the QCSE. The resulting band-flattening is directly manifested as a narrowing of the emission linewidth and an improvement in color purity.

However, the evolution of the peak wavelength (Figure 8c) reveals the competition between carrier dynamics and heat accumulation at high injection levels. At low current densities, the transferred device exhibited a shorter wavelength (blue shift) compared to the state before transfer, again consistent with the initial strain relaxation. But as the injection current increased, the pre-transfer device exhibited a typical and continuous blue shift due to the excellent heat dissipation of the bulk silicon. In comparison, the blue shift trend of the transferred device becomes less pronounced at high current densities, with the emission wavelength remaining near 510 nm. This behavior may be understood as the result of competing mechanisms under high-current injection. On one hand, increased carrier injection can weaken the QSCE in the InGaN/GaN quantum wells, leading to a blue shift of the EL peak. On the other hand, self-heating may become more pronounced in the transferred device structure, possibly due to the additional thermal resistance introduced by the bonding and interfacial layers. According to the temperature dependence of the semiconductor bandgap described by Varshni’s empirical relation, an increase in junction temperature would reduce the effective bandgap and induce a red shift in the emission peak. Such a thermally induced red shift could partially compensate for the injection-induced blue shift, resulting in the observed stalled blue shift in the peak position at higher current densities. Since temperature-dependent EL measurements, thermal simulations, and direct junction-temperature extraction were not performed in this study, this interpretation should be regarded as a plausible explanation rather than a quantitative confirmation. This observation suggests that thermal management of the bonding and interfacial layers should be considered in future high-current Micro-LED designs.

## 4. Conclusions

In summary, by employing a customized hybrid planarization strategy combining selective dry etching and iterative chemical–mechanical polishing (CMP), this study successfully achieved the wafer-level substrate transfer of 8-inch GaN-on-Si green light-emitting devices via SiO_2_–SiO_2_ direct bonding. The combined PL and wafer-warpage results suggest that the sequential removal of the massive native silicon substrate and high-stress buffer layers induces strong macroscopic wafer-shape evolution while gradually relaxing the additional compressive strain in the MQW active region. This strain relaxation increases the intrinsic bandgap of the well layer via the deformation potential effect and effectively weakens the piezoelectric polarization field in the active region to significantly mitigate the QCSE, resulting in a continuous blue shift in the emission peak and a narrowing of the spectral linewidth.

Regarding the optoelectronic performance evolution, the extreme substrate transfer and thermal annealing processes inevitably led to an increase in reverse leakage current and series resistance of the devices, while the core rectifying architecture of the p-n junction maintained robust integrity. More importantly, by eliminating the strongly light-absorbing native silicon substrate and incorporating a BSR, the transferred devices achieved a remarkable 1.9-fold enhancement in relative optical performance, showing a significant optical advantage over the original GaN-on-Si devices. However, as an inherent trade-off, the significant increase in leakage current indicates that the current transfer and processing conditions require further rigorous optimization to meet the stringent electrical demands of future micro-scale applications.

Furthermore, an analysis of the spectral evolution at high injection levels showed a stalled blue shift trend in the transferred devices. It is hypothesized that this behavior may arise from a competition between the injection-induced blue shift and potential localized self-heating caused by the additional thermal resistance of the bonding interface. This observation suggests that the thermal management of the bonded structure remains a crucial consideration for future high-current applications.

Ultimately, this work validates a high-yield wafer-level substrate transfer process for GaN-on-Si light-emitting devices and provides systematic experimental insights into stress control, optimization, and thermal management issues during the substrate transfer process. The relevant process and mechanism conclusions can also provide a reference for the subsequent fabrication and transfer of Micro-LED arrays with smaller pixel sizes.

## Data Availability

The original contributions presented in this study are included in the article. Further inquiries can be directed to the corresponding author.

## Appendix A

**Table A1:** Statistics of electrical characteristics before and after substrate transfer.

Device Group	No.	Turn-On Voltage (V)	Reverse Leakage Current at −5 V (A)
Before transfer	1	2.4	−3.962 × 10^−7^
Before transfer	2	2.6	−7.893 × 10^−8^
Before transfer	3	2.6	−2.852 × 10^−8^
Before transfer	4	2.6	−1.486 × 10^−7^
Before transfer	5	2.4	−1.578 × 10^−8^
After transfer	1	3.3	−9.870 × 10^−4^
After transfer	2	3.4	−1.719 × 10^−4^
After transfer	3	3.3	−2.970 × 10^−4^
After transfer	4	3.5	−3.410 × 10^−4^
After transfer	5	3.4	−4.228 × 10^−4^
